# The Role of IgE-Receptors in IgE-Dependent Airway Smooth Muscle Cell Remodelling

**DOI:** 10.1371/journal.pone.0056015

**Published:** 2013-02-14

**Authors:** Michael Roth, Jun Zhong, Celine Zumkeller, Chong Teck S’ng, Stephanie Goulet, Michael Tamm

**Affiliations:** 1 Pulmonary Cell Research, Department Biomedicine, University of Basel, Basel, Switzerland; 2 Pneumology, Department Internal Medicine, University Hospital Basel, Basel, Switzerland; French National Centre for Scientific Research, France

## Abstract

**Background:**

In allergic asthma, IgE increases airway remodelling but the mechanism is incompletely understood. Airway remodelling consists of two independent events increased cell numbers and enhanced extracellular matrix deposition, and the mechanism by which IgE up-regulates cell proliferation and extracellular matrix deposition by human airway smooth muscle cells in asthma is unclear.

**Objective:**

Characterise the role of the two IgE receptors and associated signalling cascades in airway smooth muscle cell remodelling.

**Methods:**

Primary human airway smooth muscle cells (8 asthmatics, 8 non-asthmatics) were stimulated with human purified antibody-activated IgE. Proliferation was determined by direct cell counts. Total collagen deposition was determined by Sircol; collagen species deposition by ELISA. IgE receptors were silenced by siRNA and mitogen activated protein kinase (MAPK) signalling was blocked by chemical inhibitors.

**Results:**

IgE dose-dependently increased extracellular matrix and collagen deposition by airway smooth muscle cells as well as their proliferation. Specifically in cells of asthma patients IgE increased the deposition of collagen-type-I, -III, –VII and fibronectin, but did not affect the deposition of collagens type-IV. IgE stimulated collagen type-I and type-VII deposition through IgE receptor-I and Erk1/2 MAPK. Proliferation and deposition of collagens type-III and fibronectin involved both IgE receptors as well as Erk1/2 and p38 MAPK. Pre-incubation (30 minutes) with Omalizumab prevented all remodelling effects completely. We observed no changes in gelatinase activity or their inhibitors.

**Conclusion & Clincal Relevance:**

Our study provides the molecular biological mechanism by which IgE increases airway remodelling in asthma through increased airway smooth muscle cell proliferation and deposition of pro-inflammatory collagens and fibronectin. Blocking IgE action prevents several aspects of airway smooth muscle cell remodelling. Our findings may explain the recently described reduction of airway wall thickness in severe asthma patients treated with humanised anti-IgE antibodies.

## Introduction

Increased IgE is a major pathology of allergic asthma which stimulates chronic inflammation and airway wall thickening leading to narrowing of the airway lumen [1], [2]. Regarding the mechanism it is unclear if the stimulating effect of IgE on airway wall remodelling is direct through the corresponding receptors or occurs indirect by increasing inflammatory mediator release from immune reactive cells or tissue forming cells [1], [2].

Airway wall remodelling consists of several independent mechanisms including (i) sub-epithelial mesenchymal cell proliferation; (ii) increased extracellular matrix (ECM) deposition; and (iii) changes of the local ECM composition [3], [4]. Recent studies indicated that airway remodelling occurs independently from inflammation and manifests much faster than suggested by earlier studies. Significant structural changes in the airway wall occurred within 8 days in volunteering patients with mild asthma in response to inhaled allergens or to cholinergic stimuli [5]. In asthma patients long term therapy with humanised anti-IgE antibodies significantly reduced the thickness of the airway wall and of the reticular basement membrane within 6 and 12 months [6], [7]. Since this beneficial clinical effect of anti-IgE antibody therapy was independent of eosinophil infiltration the mechanism behind the reduced airway wall thickness remained unclear [6], [7]. It was suggested that in humans IgE may have a direct effect on airway wall remodelling, while earlier animal studies implicated indirect effects of IgE on airway remodelling, through stimulating the release of cytokines and growth factors from immune-reactive cells [8]–[10]. Unfortunately, none of these studies dissected the role of the two IgE receptors, Igε−RI and Igε−RII (CD23) in airway remodelling. Thus, this raises the question if anti-IgE antibody therapy in long term can prevent or reduce airway remodelling [11].

The thickening of the airway wall in asthma is largely caused by hypertrophy and hyperplasia of airway smooth muscle cells (ASMC) which express and respond to the Igε−RI and Igε−RII [12]–[14].

There is evidence that allergens and IgE can penetrate through the basement membrane towards tissue forming cells and activate them. In asthma, the function of the epithelium as a barrier is deranged and thus may allow allergens to get into direct contact with ASMC [15]–[18]. Furthermore, some allergens digest the ECM of the basement membrane leading to local inflammation and blood vessel leakage [15], [18]. The local modification of the ECM composition changes the function and differentiation of tissue forming cells in asthma [19]. Since the sub-epithelial fibroblasts and ASMC are the major producers of ECM of the airway wall a modification of its composition through matrix metalloproteinases (MMP) and their inhibitors (TIMP) will have a feedback mechanism on the cells function [20], [21]. In isolated ASMC the presence of asthma patient’s serum specifically increased the deposition of fibronectin, perlecan, laminin, and chondroitin sulphate which together create a pro-inflammatory and mitogenic condition leading to persistent inflammation [22]. In this context it should be noted that increasing the deposition of ECM components does not need to increase their gene transcription; existing ECM can be re-arranged by specific enzymes and precursors of soluble collagens and fibronectin can be integrated into the existing ECM meshwork [23], [24].

Therefore, we investigated the direct effect of purified human IgE on proliferation, ECM and collagen species deposition in ASMC derived from asthma and non-asthma patients.

## Materials and Methods

### Cell Culture

**Figure 1:**
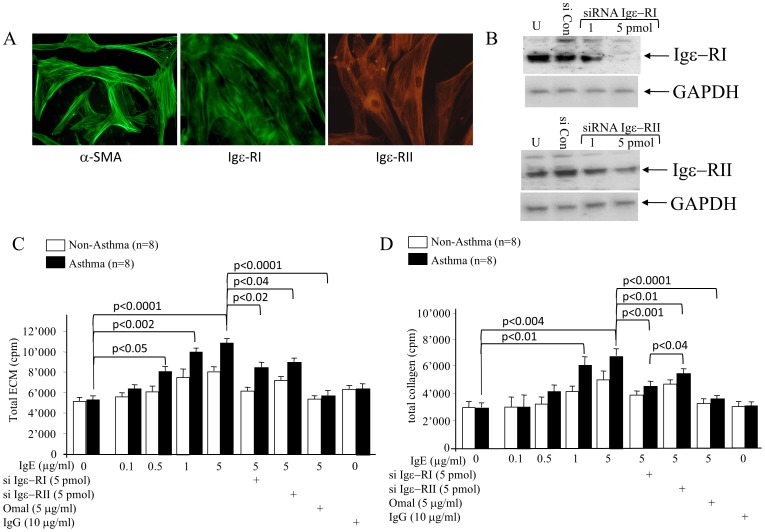
Cell characterisation and the effect of IgE and its receptors on ECM deposition. (A) ASMC immunofluorescence staining for filamentous α-SMA, Igε-RI and Igε-RII. (B) Immune-blot of Igε-RI and Igε-RII siRNA treatment (48 hours) on protein expression; siCon: scrambled siRNA, U: untreated cells, GAPDH: house-keeping protein. (C) IgE induced ECM. (D) Collagen deposition (48 hours) and its modulation by siRNA targeting Igε-RI, Igε-RII or by Omalizumab. IgG served as control. Statistics: ANOVA. siCon: control siRNA; U: untreated cells.

Bronchial biopsies were obtained from 16 patients (8 mild/moderate allergic asthma; 8 non-asthma/non-atopic) after written consent and approval by the local ethic committee of both Basel, Switzerland (Ref. Nr.: EK 05/06). None of the participants used any anti-asthma therapy for 1 week prior to the study. ASMC were isolated as described earlier [20]. All cells were characterised by positive immune-staining for filamentous α-smooth muscle cell actin and the expression of Igε−RI and Igε−RII was confirmed by immuno-staining (Igε−RI sc-68942; Igε−RII sc-70592: Santa Cruz Biotechnology, Santa Cruz, USA) as shown in figure 1A.

### IgE Stimulation

Highly purified human IgE (Diatek, Oslo, Norway) was incubated overnight with an activating anti-IgE antibody before stimulating ASMC for 24, 48 or 72 hours. Purified human IgG treatment (Diatek) served as control. Prior to experiments confluent ASMC were serum starved for 24 hours. IgE activity was blocked by the humanized clinically used anti-IgE antibody Omalizumab (Novartis, Basel, Switzerland).

### ECM Composition

The ECM composition was analysed by ELISA by a modified procedure published earlier [25]. Confluent ASMC were treated for 48 or72 hours with TGF-β1, IgE or signalling inhibitors before being washed 3× with phosphate buffered saline (PBS) and fixation in 2% formalin in PBS (10 minutes). Unspecific antibody binding was blocked by 30 minutes incubation in 1% bovine serum albumin, followed by overnight (4°C) incubation with antibodies to collagen types-I, -III, -IV, –VII or fibronectin (all Santa Cruz Biotech). After 3 washes with PBS, the secondary antibodies were added and incubated (1 hour, room temperature). After 3 washes (PBS), 100 µl of peroxidase-conjugated streptavidin (1∶500) was added per well (30 minutes). The substrate 2,2'-Azino-*bis*-(3-ethylbenzo thiazoline)-6-sulfonic acid was added for 20 minutes and stopped by 0.3N H_2_SO_4_. The absorbance (405/490 nm) was read with ELISA plate reader (Model 3550; Bio-Rad, Inc., Hercules, CA) [18].

### Deposition of Total ECM and Collagens

The deposition of total ECM and collagens was determined by [^3^H]-proline incorporation in confluent ASMC as described earlier [25].

### MAPK and IgE-receptor Inhibition by siRNA

MAPK Erk1/2 and p38 were inhibited with 0, 1, or 5 µM of either SB203580 (p38 MAPK) or PD98059 (Erk1/2: Calbiochem, Basel, Switzerland) for 24 hours before being stimulated.

To study the role of the two IgE receptors on IgE-induced ECM deposition, cells were pre-treated with 0, 1, or 5 µM of IgE receptor specific siRNAs (Santa Cruz Biotech) for 24 hours. After IgE stimulation, half the concentration of the siRNAs was added every 24 hours.

### RNA Extraction and Real Time PCR

Total RNA was extracted using a Qiagen kit following the instructions of the distributor. Real time PCR was performed for Col1A1, Col3A1, Col4A1, Col7A1 and fibronectin using the primers and conditions listed in table 1.

**Table 1:** Expression of mRNA encoding procollagens and fibronectin in IgE stimulated ASMC.

	Day 0	Day 2	Day 4
Pro-collagen1A1	12.34±0.64	14.52±1.12	12.15±0.54
Pro-collagen3A1	4.51±0.32	3.84±0.83	5.62±0.31
Pro-collagen4A1	8.36±0.38	9.38±0.53	7.46±0.63
Pro-collagen7A1	4.23±0.19	3.29±0.25	5.34±0.41
Fibronectin	13.15±.78	16.39±0.35	14.62±0.97

### Cell Count

Cells were seeded at a density of 1×10^5^/cm^2^ and serum deprived for 24 hours before stimulation. After 2 days, the cells were harvested and manually counted in a hematocytometer (improved Neugebaur) [26].

### Statistics

The Null-hypothesis was that neither IgE nor Omalizumab affected ECM deposition and composition. Data analysis was performed with ANOVA (Bonferroni correction) and p<0.05 was regarded as significant.

## Results

In figure 1A, we confirmed that the ASMC which we isolated from asthma and non-asthma patient airways express Igε-RI and Igε-RII as well as filamentous α-SMA. Pre-treatment (48 hrs) with IgE receptor specific siRNAs depleted Igε-RI and Igε-RII protein expression significantly (figure 1B).

The deposition of total ECM and of total collagens was dose dependently increased by IgE over 48 hours (Figure 1C, 1D). IgG stimulation had no effect on ECM or collagen deposition by ASMC (data not shown). Down regulation of Igε-RI indicated its dominant role in ECM deposition (Figure 1C). Pre-treatment (30 minutes) of cells with the anti-IgE antibody Omalizumab completely prevented IgE-induced ECM deposition (Figure 1C). Total collagens were specifically up-regulated by IgE in asthma patient’s ASMC, and inhibition of Igε-RI reduced collagen deposition more effectively than down regulation of Igε-RII, while prevention of IgE binding by Omalizumab completely inhibited collagen deposition (Figure 1D). IgG (10 µg/ml) had no effect on total ECM or total collagen deposition (Figure 1C, D).

**Figure 2:**
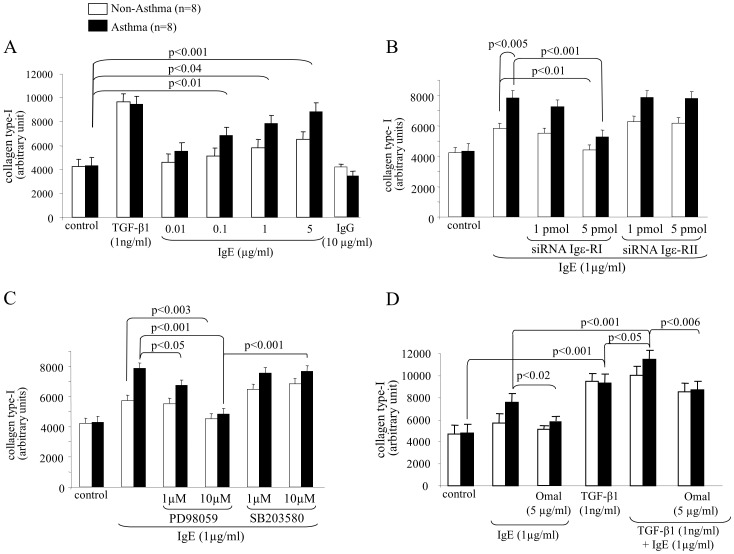
IgE induces collagen type-I deposition by ASMC through Igε-RI and Erk1/2 MAPK. (A) Induction of collagen type-I deposition by IgE by ASMC and (B) the mediator role of Igε-RI and Igε-RII. (C) Contribution of MAPK to Igε-RI and Igε-RII signalling on IgE induced collagen type-I deposition by ASMC. (D) Specificity of IgE neutralising antibody treatment on combined TGF-β1 and IgE collagen type-I deposition by ASMC.

Investigating the effect of IgE on different collagen types we observed a dose dependent significant increase of collagen type-I deposition by ASMC of asthma patients, while the effect on non-asthma patient cells was less strong (Figure 2A). TGF-β1 (positive control) induced, but did show any disease specific effect; as IgG (negative control) did not affect collagen type-I deposition (Figure 2A). Pre-treatment with siRNA showed that Igε-RI mediated the stimulating effect of IgE on collagen type-I deposition, while Igε-RII was not involved (figure 2B). Regarding MAPK signalling ERK1/2 mediated IgE induced collagen type-I deposition, while p38 MAPK did not (Figure 2C). In figure 2D, we provide evidence that IgE combined with TGF-β1 had an additive effect on collagen type-I deposition, which was prevented by Omalizumab; however, the IgE antibody had no reducing effect on TGF-β1 induced collagen type-I deposition.

**Figure 3:**
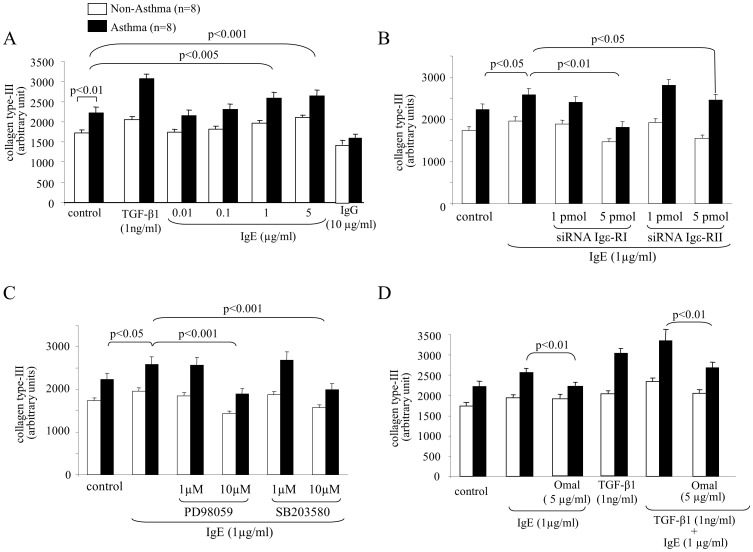
Collagen type-III deposition by IgE is regulated through the Igε-RI and Erk1/2 and p38 MAPK. (A) Induction of collagen type-III deposition by IgE by ASMC and (B) the mediator role of Igε-RI and Igε-RII. (C) Contribution of MAPK to Igε-RI and Igε-RII signalling on IgE induced collagen type-III deposition by ASMC. (D) Specificity of IgE neutralising antibody treatment on combined TGF-β1 and IgE collagen type-III deposition by ASMC.

Collagen type-III deposition was higher in cells from asthma patients compared to controls, which was further increased by TGF-β1 and IgE stimulation (Figure 3A). IgG did not induce collagen type-III deposition (Figure 3A). Igε-RI mediated the major effect of IgE on collagen type-III deposition while Igε-RII played a lesser role (Figure 3B). MAPK studies indicated that both ERK1/2 and p38 were needed for IgE induced collagen type-III deposition (Figure 3C). In cells from asthma patients, the combination of TGF-β1 with IgE resulted in an additive increased deposition of collagen type-III and the IgE part was blocked by Omalizumab (Figure 3D).

**Figure 4:**
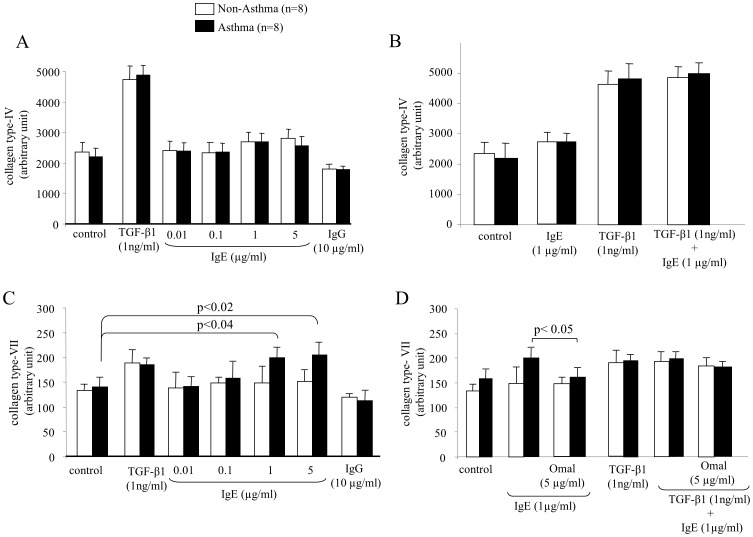
IgE does not increase collagen type-IV deposition, but increases collagen type-VII diseases specifically. (A) IgE does not induce collagen type-IV deposition by ASMC. (B) IgE does not modify TGF-β1-induced collagen type-IV deposition. (C) IgE disease specifically increases the deposition of collagen type-VII by ASMC of asthma patients, (D) but has no additive effect on TGF-β1-induced collagen type-VII deposition.

IgE did not up-regulate collagen type-IV deposition, while TGF-β1 doubled its deposition, while IgG had no effect (Figure 4A). Combined IgE did not add to the stimulatory effect of TGF-β1 on collagen type-IV deposition (Figure 4B).

Collagen type-VII deposition was stimulated by TGF-β1; but for IgE only the two highest concentrations up-regulated its deposition by ASMC of asthma patients only, IgG had no effect (Figure 4C). Pre-incubation of the cells with anti-IgE antibody significantly reduced IgE dependent collagen type-VII deposition by asthma cells but had no effect on TGF-β1 (Figure 4D). Due to the high variability of collagen type-VII deposition, we did not observe a significant additive effect when TGF-β1 was combined with IgE (Figure 4D).

**Figure 5:**
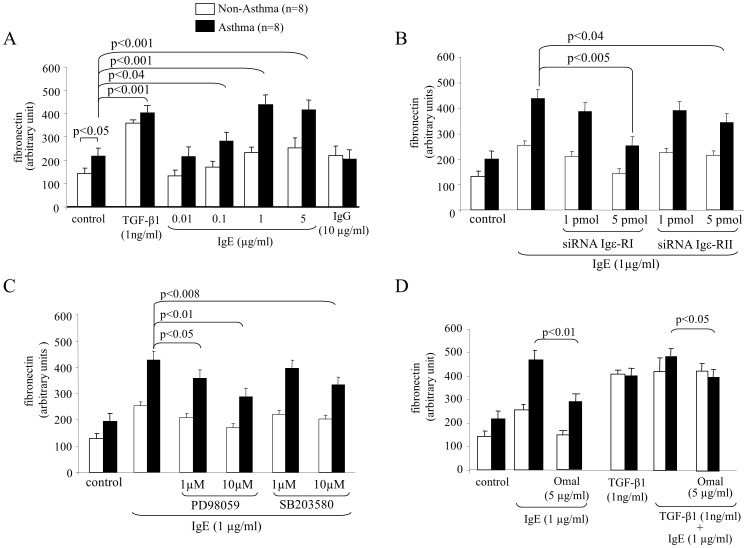
Fibronectin deposition by IgE requires both IgE receptors and Erk1/2 and p38 MAPK. (A) Induction of fibronectin deposition by IgE by ASMC and (B) the mediator role of Igε-RI and Igε-RII. (C) Contribution of MAPK to Igε-RI and Igε-RII signalling on IgE induced fibronectin deposition by ASMC. (D) Specificity of IgE neutralising antibody treatment on combined TGF-β1 and IgE fibronectin deposition by ASMC.

Based on earlier studies with human serum containing high levels of IgE, we investigated its effect on fibronectin deposition. As shown in figure 5A, ASMC of asthma patient produced significantly higher basal levels of fibronectin compared to controls. Stimulated with TGF-β1 fibronectin levels were comparable (Figure 5A). IgE increased fibronectin deposition in a dose dependent and disease specific pattern reaching a plateau of 1µg/ml, while IgG had no effect (Figure 5A). Compared to Igε-RII our data showed that Igε-RI mediated most of the IgE effect on fibronectin deposition (Figure 5B). Like for collagen type-III, both MAPKs ERK1/2 and p38 were involved in the IgE induced deposition of fibronectin (Figure 5C). When combined, IgE further increased TGF-β1 induced fibronectin deposition, however, the effect was less than additive and was inhibited by Omalizumab (Figure 5D).

**Figure 6:**
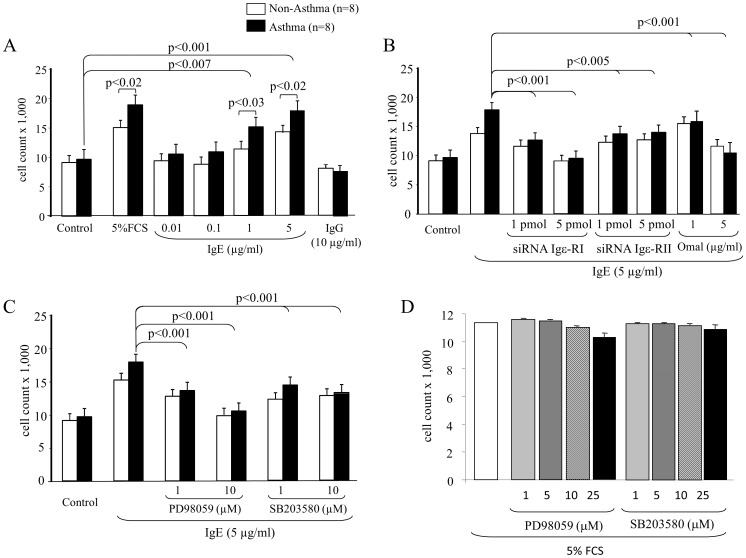
IgE-induced ASMC proliferation needs both IgE receptors and Erk1/2 and p38 MAPK. (A) The dose-dependent effect of IgE on ASMC proliferation over 48 hours determined by manual cell counts. (B) The role of IgE-receptors on IgE-induced ASMC proliferation. (C) MAPK signalling in IgE-induced ASMC proliferation. Bars represent the mean ± S.E.M. of six asthma ASMC and seven non-asthma ASMC lines. All experiments were performed in triplicate. (D) Viability of ASMC in the presence of increasing concentrations of MAPK inhibitors PD98059 and SB203580. Experiments were performed in triplicate in four ASMC lines and data represent the mean ± S.E.M.

Finally, we observed that IgE dose-dependently stimulated the proliferation of ASMC, with a significantly stronger effect on ASMC of asthma patients at concentrations >1 µg/ml (Figure 6A). FCS (5%) induced a similar disease specific increase of cell numbers in asthmatic ASMC as IgE, while IgG had no such effect (Figure 6A). The role of the two IgE receptors on proliferation control is shown in figure 6B and revealed that Igε-RI was more important for proliferation control than Igε-RII; however, the inhibition of both receptors was necessary to prevent IgE-induced ASMC proliferation (Figure 6B). Our data implied that ERK1/2 MAPK played a more important role in IgE dependent ASMC proliferation than p38 MAPK; however, the blockade of p38 MAPK also significantly reduced proliferation (Figure 6C). In figure 6D we provide data showing that the two MAPK inhibitors did not significantly reduce ASMC viability over 48 hours at the concentrations used for signalling inhibition.

As shown in table 1 the stimulating effect of IgE on collagens and fibronectin deposition could not be explained by increased transcription of the corresponding genes. Even so IgE had a mild stimulating effect on mRNA transcript numbers for pro-collagenA1A type-I and III, this effect was very small compared to that of TGF-β1 and did not parallel the increased deposition described above.

## Discussion and Conclusions

This study presents further evidence that IgE directly activates ASMC and thereby modifies the composition of the ECM during airway wall remodelling. Our data indicate that the IgE induces the deposition of the pro-inflammatory collagens type-I and fibronectin mainly independent of up-regulating transcription of the corresponding gens. This effect of IgE on ASMC is mainly mediated through Igε-RI and ERK1/2 MAPK activity. The same signalling pathway also controls IgE-induced ASMC proliferation. Despite the observation that Igε-RI is the major IgE receptor, Igε-RII and p38 MAPK also contribute to airway remodelling, however, to a much lower extend.

The hypothesis that IgE actively induces airway wall remodelling has recently being supported by two clinical studies performed in patients with severe asthma [6], [7]. Blocking IgE significantly reduced the thickening of the airway wall and airway inflammation and reduced inflammation in both studies. Taking into account that the daily turnover of the ECM in the healthy human lung was estimated with 10% [27] and that collagen deposition increased by more than 3 fold within 8 days of HDM challenge in mild asthmatic airways^3^ indicates that airway remodelling is a much faster and more flexible pathology than expected. Airway remodelling in asthma was earlier linked to a disease specific pathological structure and function of the airway wall forming cells and ECM composition [28], [29]. We have shown earlier that ASMC isolated from asthma patients produce a different ECM composition than ASMC from non-asthmatic patients and also respond more profoundly to remodelling stimuli such as TGF-β1 and connective tissue growth factor [30], [31]. Our here described finding, that IgE especially increases the ratio of the deposition of pro-inflammatory collagen type-I and type-III is paralleled by a recent in vivo study of airway wall [32].

Collagen type-I increased ASMC proliferation, while it is not clear if it alters ASMC contractility [20], [28]. Importantly, collagen type-I in asthmatic airways reduced ASMC response to steroids which may explain a certain type of steroid resistance in asthma patients [33], [34]. Collagen type-I may also explain different response of asthma patients cells on the interaction of the plasminogen activator and tensacin-C [35], [36].

The content of collagen type-III in airway wall was increased in childhood and adult asthma; but only in adults, the ratio of collagen type-III in the airways is correlated with severity [22], [37]. Unfortunately, steroid therapy did not reduce asthma associated deposition of collagen type-I or type-III [37]. This may due to the increased resistance to steroids in a collagen rich environment or to the inability of steroids to down regulate collagens during inflammation [32]. In house dust mite induced mouse asthma transilast, a mast cell inhibitor, down regulated collagen type-I and –III deposition [38]. Most studies agree that the content of collagen type-IV is not significantly modified in chronic airway inflammation, which is in line with our data showing that IgE did not significantly alter collagen type-IV deposition; however, TGF-β1 did [16], [32]–[34].

In the context of airway inflammation, the role of collagen type-VII has not been studied well. In a rhesus monkey model inhaled house dust mite allergens increased the deposition of collagen type-VII in the basement membrane which was linked to the loss of epithelial cell integrity [18]. In other inflammatory diseases with disrupted basement membrane structure collagen type-VII deposition increased parallel to inflammation and reduced cell–cell adhesion and epithelial cell function [39]. Our data indicate that ASMC are able to produce and depose collagen type-VII which was thought to be produced mainly be epithelial cells. However, when compared to the other collagens the total amount of collagen type-VII deposed by ASMC seems to be small.

The role of fibronectin in asthma is controversial. Two studies analysed the *in vivo* deposition of fibronectin in asthmatic airway and found either no change or even a reduction [32], [40]. In contrast, isolated fibroblasts, myo-fibroblasts and ASMC of asthma patients deposed more fibronectin than controls and mediated the response to mast cells, allergens and epithelial cell to mesenchymal cell transition [41]–[44].

Our study indicates that the major effect of IgE on remodelling is mediated via Igε-RI and ERK1/2 MAPK and explains the increase of collagen type-I and –III deposition as well as of proliferation. In other cell types, including epithelial and mast cells, IgεR-I activated both Erk1/2 and p38 MAPK [45], [46]. In lymphocytes, IgE activated p38 MAPK signalling, but not Erk1/2 or JNK [47]. Comparable to our study, serum of severe asthmatics, which contained high IgE levels, increased total ECM deposition by ASMC and the up regulation of fibronectin deposition involved p38 MAPK [30].

Our data indicate that anti-IgE therapy may have a reductive effect on airway remodelling by reducing the *de novo* synthesis and deposition of pro-inflammatory collagen types in the airways. If this observation could be confirmed in long term therapy with anti-IgE antibodies, it may explain the clinically documented improvement of lung function.
